# Application of a loop-mediated isothermal amplification (LAMP) assay targeting *cox1* gene for the detection of *Clonorchis sinensis* in human fecal samples

**DOI:** 10.1371/journal.pntd.0005995

**Published:** 2017-10-09

**Authors:** S. M. Mazidur Rahman, Hyun Beom Song, Yan Jin, Jin-Kyoung Oh, Min Kyung Lim, Sung-Tae Hong, Min-Ho Choi

**Affiliations:** 1 Department of Parasitology and Tropical Medicine, Seoul National University College of Medicine, and Institute of Endemic Diseases, Seoul National University Medical Research Center, Seoul, Korea; 2 Department of Microbiology, Dongguk University School of Medicine, Gyeongju, Korea; 3 National Cancer Center, Graduate School of Cancer Science and Policy, Goyang, Gyonggi, Korea; University of California Berkeley, UNITED STATES

## Abstract

**Background:**

Clonorchiasis is prevalent in the Far East, and a major health problem in endemic areas. Infected persons may experience, if not treated, serious complications such as bile stone formation, pyogenic cholangitis, and even cholangiocarcinoma. Early diagnosis and treatment are important to prevent serious complications and, therefore, the simple and reliable diagnostic method is necessary to control clonorchiasis in endemic areas, where resources for the diagnosis are limited.

**Methodology/Principle findings:**

The loop-mediated isothermal amplification (LAMP) assay has been applied for the detection of *Clonorchis sinensis* DNA. Six primers targeting eight locations on the cytochrome c oxidase subunit 1 gene of *C*. *sinensis* were designed for species-specific amplification using the LAMP assay. The LAMP assay was sensitive enough to detect as little as 100 fg of *C*. *sinensis* genomic DNA and the detection limit in 100 mg of stool was as low as one egg. The assay was highly specific because no cross-reactivity was observed with the DNA of other helminths, protozoa or *Escherichia coli*. Then, LAMP assay was applied to human fecal samples collected from an endemic area of clonorchiasis in Korea. Using samples showing consistent results by both Kato-Katz method and real-time PCR as reference standards, the LAMP assay showed 97.1% (95% CI, 90.1–99.2) of sensitivity and 100% (95% CI, 92.9–100) of specificity. In stool samples with more than 100 eggs per gram of feces, the sensitivity achieved 100%.

**Conclusions:**

To detect *C*. *sinensis* in human fecal samples, the LAMP assay was applied and achieved high sensitivity and specificity. The LAMP assay can be utilized in field laboratories as a powerful tool for diagnosis and epidemiological survey of clonorchiasis.

## Author summary

Clonorchiasis, a parasitic infection by *Clonorchis sinensis*, is one of the major public health problems in eastern Asian countries, including China, Korea, and Vietnam, causing cholangiocarcinoma if not treated. The definitive diagnosis is important for successful treatment and prevention of the infection in endemic areas. The fecal examination is a standard diagnostic method and mainly based on microscopic observation of eggs in feces. However, they sometimes miss lightly infected cases and can lead to misidentification at the species level because of morphological similarity between the eggs of liver flukes and minute intestinal flukes. To overcome the diagnostic limitations of stool examination, ELISAs and PCRs have been developed as a sensitive and specific diagnostic method, but they require sophisticated equipment such as thermal cycler and spectrophotometry, which has prevented the widespread use of these techniques in low resource areas. We developed a LAMP assay targeting the cytochrome c oxidase subunit 1 gene of *C*. *sinensis* for rapid and simple detection of *C*. *sinensis* DNA in human fecal samples. The LAMP assay was highly specific and sensitive enough to detect as little as 100 fg of *C*. *sinensis* genomic DNA and to detect a single egg of *C*. *sinensis* in 100 mg of stool samples. Then, LAMP assay was applied to human fecal samples collected from an endemic area of clonorchiasis in Korea. Using samples showing consistent results by both Kato-Katz method and real-time PCR as reference standards, the LAMP assay showed 97.1% (95% CI, 90.1–99.2) of sensitivity and 100% (95% CI, 92.9–100) of specificity. In stool samples with more than 100 eggs per gram of feces, the sensitivity achieved 100%. Due to the sensitive and specific detection of *C*. *sinensis* DNA in fecal samples, the LAMP assay can be applied in field laboratories as a powerful tool for diagnosis and epidemiological survey of clonorchiasis.

## Introduction

Clonorchiasis is an important human parasitic infection and is highly prevalent in eastern Asian countries, including China, Korea, and Vietnam [1, 2]. In Korea, the *Clonorchis sinensis* egg positive rate in the general population is 1.9%, and approximately 1 million people are estimated to be infected [3]. However, in some endemic provinces and river basins, the infection rate is reported to be more than 10% [4]. Most infected people have no symptoms, but chronic infection induces some clinical manifestations, including abdominal or epigastric discomfort, fatigue, jaundice, vomiting, fever, and diarrhea [5]. The most important and serious complication of *C*. *sinensis* infection is cholangiocarcinoma, and the parasite has been classified as a group 1 biological carcinogen [6].

The specific diagnosis of *C*. *sinensis* is important for successful treatment and control of the infection. The Kato-Katz (KK) method and/or formalin-ether concentration technique are commonly used for clonorchiasis diagnosis [7]. However, stool examinations are not highly effective because lightly infected cases can be missed [7]. Moreover, due to morphological similarities, the eggs of *C*. *sinensis* are easily confused with the eggs of other flukes (e.g., Heterophyidae or Opisthorchiidae). For this reason, specific diagnosis of *C*. *sinensis* eggs in the feces is sometimes difficult by KK method especially for the lightly infected cases [1]. ELISAs for serodiagnosis of clonorchiasis are widely used, but they cannot differentiate between past and current infections [8, 9]. Recently, a sensitive, specific ELISA has been developed to detect *C*. *sinensis* antigens directly from stool samples, but its application in the field has not yet been evaluated [10]. Several PCR assays have been developed to detect *C*. *sinensis* DNA in stools, but the sensitivities and specificities of these PCR assays vary depending on their target genes [11–16]. Moreover, these PCR methods require sophisticated equipment, such as a thermal cycler, which has prevented the widespread use of these techniques in low resource areas.

An alternative DNA amplification technique known as loop-mediated isothermal amplification (LAMP) has been developed [17]. The LAMP assay can be performed under isothermal conditions (60°C to 65°C); therefore, a simple water bath or block heater is sufficient to amplify the specific DNA [17]. Moreover, the assay allows visual detection of DNA amplification through the addition of fluorescent dyes [18]. It has several advantages over conventional PCRs such as higher sensitivity and specificity, rapid and simple procedures, and is a good candidate approach to detect many pathogens including parasites in field conditions, without the use of expensive equipment [19]. Shorter reaction time with visual judgment of positivity without requiring sophisticated equipments makes it an attractive diagnostic method for field application.

The LAMP assay has been applied successfully to detect various parasitic infections, including opisthorchiasis [20–22], schistosomiasis [23], paragonimiasis [24], fascioliasis [25], and taeniasis [26]. Recently, a LAMP technique has been developed to detect *C*. *sinensis* DNA in freshwater snails [27] and fish [19], as a tool of control and prevention of clonorchiasis in endemic areas; however, there is still no report of *C*. *sinensis* DNA detection in human fecal samples.

In the present study, a highly sensitive and specific LAMP assay has been developed to detect *C*. *sinensis* DNA in human stool samples. The LAMP assay was evaluated using the fecal samples collected from a clonorchiasis endemic area of Korea and compared with the combined results of KK method and real-time PCR.

## Materials and methods

### Ethics statement

The design of this study was reviewed by the institutional review board of Seoul National University Hospital (IRB approval number E-1512-075-727). Institutional review of this study was waivered because this study used anonymized stool samples that were randomly selected from the pool of stool samples of the residents of an endemic area of clonorchiasis in Korea, which had been obtained from the previous studies [28, 29]. The animal experiment was reviewed and approved by the Institutional Animal Care and Use Committee (IACUC) of Seoul National University, Seoul, Korea and followed the National Institutes of Health (NIH) guideline for the care and use of laboratory animals (ISBN 0-309-05377-3).

### Adult worms of *C*. *sinensis*

Metacercariae of *C*. *sinensis* were collected from naturally infected freshwater fishes in Korea. Adult worms were recovered from the bile ducts of Sprague-Dawley rats after 2 months of infection with metacercariae. DNA was extracted from adult worms and used as a positive control for the LAMP assay.

### Stool samples

For analysis of the LAMP assay, stool samples were randomly selected from the pool of stool samples of the residents of Sancheong county in Korea, where clonorchiasis is endemic, and risk factors and incidence of cholangiocarcinoma among this resident were investigated since 2006 [28, 29]. For each stool sample, two KK smears and one real-time PCR were performed. For the KK smear, 41.7 mg of feces was examined by microscopy and multiplied by 24 to convert to eggs per gram of feces (EPG) [7]. Specific diagnosis of *C*. *sinensis* eggs in the feces is sometimes difficult by KK method due to the morphological similarities of eggs of other flukes [1]. To confirm whether the infection is due to the *C*. *sinensis*, in addition to KK, our laboratory-developed sensitive and specific real-time PCR was performed according to procedures previously described [16]. The real-time PCR reaction was performed in 4 μl of extracted stool DNA in a total volume of 25 μl. Then, stool samples positive by both method were considered as a positive reference sample and negative samples by both method were considered as a negative reference sample. Based on the results of two KK smears and one real-time PCR for each stool sample, 70 *C*. *sinensis*-positive and 50 *C*. *sinensis*-negative samples were selected. The selected samples were from 120 subjects (age range, 31–80 years; median age, 60.5 years; 71 males). By evaluating these samples with the LAMP assay, the diagnostic accuracy of the assay was compared with the combined results of KK method and real-time PCR.

### Extraction of DNA

The genomic DNA from adult *C*. *sinensis* and other parasites was extracted using QIAamp Tissue Kit (QIAgen) following the manufacturer’s instruction. DNA from stool samples was extracted using the procedures previously described [30]. Briefly, 100 mg stool was washed twice with 1 ml PBS and centrifuged at 8000 rpm for 5 min. After centrifugation, the pellet was resuspended in 200 μl of a 2% polyvinylpolypyrolidone (Sigma, St. Louis, MO, USA) solution and then heated in a heat block at 100°C for 10 min. After treatment with sodium dodecyl sulfate–proteinase K for 2 hr at 55°C, the DNA was eluted by adding 100 μl elution buffer through QIAamp Tissue Kit spin columns (Qiagen, Hilden, Germany) according to the manufacturer’s instructions.

### Primers for the LAMP assay

**Fig 1:**
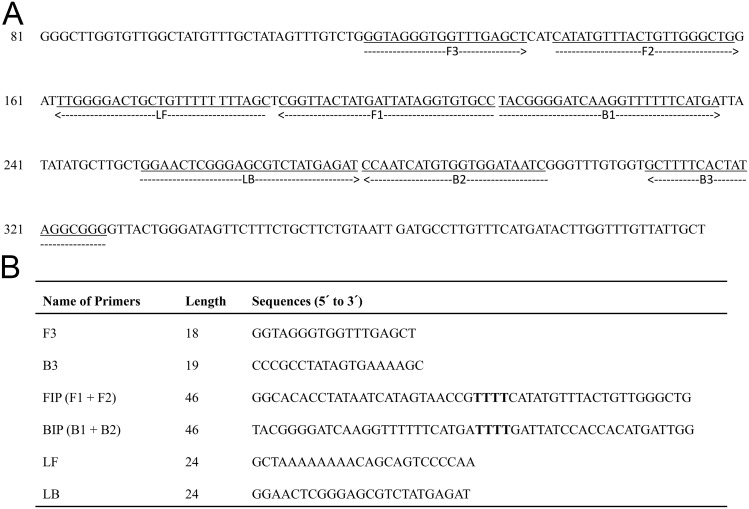
Primer sets used for amplification of the *Clonorchis sinensis* cytochrome c oxidase subunit 1 (*cox1*) gene by the loop-mediated isothermal amplification (LAMP) technique. (A) Locations of the primer sequences. (B) Names, length and sequences of six primers. F3 and B3 represent forward and backward external primers, respectively; FIP and BIP represent forward and backward internal primers, respectively; and LF and LB represent forward and backward loop primers, respectively. Primer FIP consists of F1 complementary sequence and F2 direct sequence. Primer BIP consists of B1 direct sequence and B2 complementary sequence.

Sets of forward and backward external primers (F3 and B3), forward and backward internal primers (FIP and BIP) and forward and backward loop primers (LF and LB) were designed based on the sequence of the cytochrome c oxidase subunit 1 (*cox1*) gene of *C*. *sinensis* (GenBank accession no. AF181889). The LAMP primers were designed using the software ‘PrimerExplorer V4’ (http://primerexplorer.jp/e). The list of primers and their locations in *cox1* gene are shown in Fig 1.

### The LAMP reaction

The LAMP assay was performed in a total reaction mixture volume of 25 μl, containing 2.5 μl of 10x ThermoPol reaction buffer (New England BioLabs, Ipswich, MA, USA), 6 mM MgSO_4_, 1 M betaine, 1.4 mM dNTP mix, 0.2 μM each of F3 and B3 primers, 1.6 μM each of FIP and BIP primers, 0.8 μM each of LF and LB primers, 8 U *Bst* DNA polymerase (New England BioLabs) and 4 μl of the template DNA. The reaction mixture was incubated at 64°C for 60 min in a heat block and followed by incubation at 82°C for 2 min to terminate the reaction.

Amplified LAMP products were detected by adding 1.0 μl of 1:10 diluted 10,000x concentration of SYBR Green I (Invitrogen, Carlsbad, CA, USA) to each tube. The amplicon was observed directly either by the naked eye or by placing the reaction tube under UV light (Gel documentation system, UVItech, Cambridge, UK). In addition, 5.0 μl of the LAMP products was examined by electrophoresis on a 2% agarose gel, followed by ethidium bromide staining and visualization under UV light.

### Sensitivity and specificity of the LAMP assay

Sensitivity of the assay was determined by amplifying 10-fold serial dilutions of genomic DNA of the *C*. *sinensis* adult worm from 1 ng to 1 fg. Also, egg-negative feces were experimentally spiked with 10,000, 1,000, 100, 10 and 1 egg(s) of *C*. *sinensis*. Eggs were collected by tearing the uterus of adult *C*. *sinensis* under a stereomicroscope. Numbers of eggs were counted and 10-fold serial dilutions were made from 10,000 to 100 eggs. For the accuracy of the assay, 10 and 1 egg(s) were collected under a stereomicroscope using fine tip glass Pasteur pipette and spiked in the negative feces. DNA was extracted from each spiked feces and amplified by the LAMP assay for determination of the minimum detectable number of *C*. *sinensis* eggs in feces.

Specificity of the LAMP assay was evaluated using DNA isolated from trematodes (*Metagonimus yokogawai*, *Opisthorchis viverrini* and *Fasciola gigantica*), cestodes (*Spirometra erinacei* and *Diphyllobothrium latum*), nematodes (*Ascaris lumbricoides*, *Ascaris suum*, *Necator americanus* and *Trichuris trichiura*), protozoa (*Cryptosporidium parvum*, *Entamoeba histolytica* and *Giardia lamblia*) and *Escherichia coli*. For each reaction, the same amount of DNA, 1 ng, was utilized.

### PCR with external primers F3 and B3

PCR was performed with two LAMP external primers (F3 and B3) to compare the sensitivity to that of LAMP and to confirm that LAMP correctly amplified the target. The PCR reaction was conducted in a 25 μl reaction mixture containing 2.5 μl of 10x PCR buffer, 0.2 mM dNTP mix, 0.4 μM each of F3 and B3 primers, 1.5 U *Ex Taq* polymerase (Takara, Otsu, Shiga, Japan) and 4 μl of the template DNA. Ten-fold serial dilutions of *C*. *sinensis* genomic DNA, starting from 1 ng down to 1 fg, were used as PCR templates.

## Results

**Fig 2:**
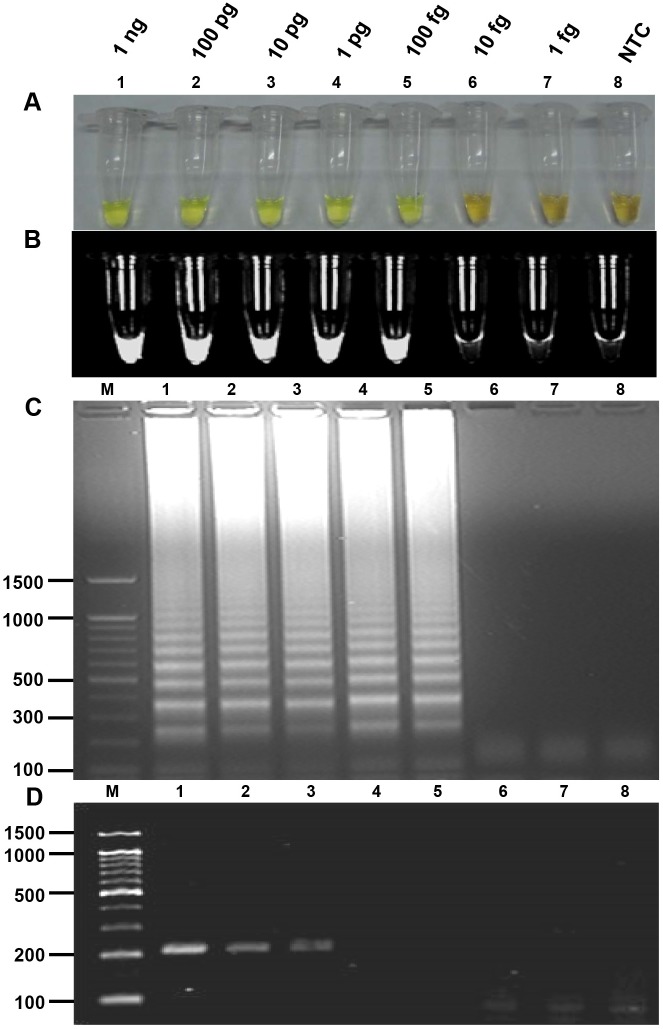
Sensitivity of the loop-mediated isothermal amplification (LAMP) assay and PCR for the detection of *Clonorchis sinensis* genomic DNA. Ten-fold serial dilutions starting from 1 ng of genomic DNA (lane 1) down to 1 fg (lane 7) were tested. (A) Naked eye detection of LAMP products using SYBR Green I. A green color indicates a positive reaction, and an orange color indicates a negative reaction. (B) Fluorescence of LAMP products after using SYBR Green I followed by detection under UV light. (C) Agarose gel electrophoresis of LAMP products followed by ethidium bromide staining and detection under UV light. (D) PCR with outer primers F3 and B3. Values in the left are in base pairs. Lane 8, non-template control (NTC); lane M, molecular marker.

LAMP products were visually detected by the naked eye after adding SYBR Green I dye to each reaction tube. The color of the reaction solution was green in the presence of *C*. *sinensis* DNA (positive LAMP reaction); otherwise, in the absence of *C*. *sinensis* DNA, it remained orange (negative reaction) (Fig 2A). The LAMP products were also visualized by placing the reaction tube under UV light (Fig 2B). Upon gel electrophoresis, the LAMP products were observed as typical ladder-like bands (Fig 2C). When PCR was performed using *C*. *sinensis* genomic DNA with F3 and B3 primers, an expected PCR product of 210 bp was obtained (Fig 2D).

**Fig 3:**
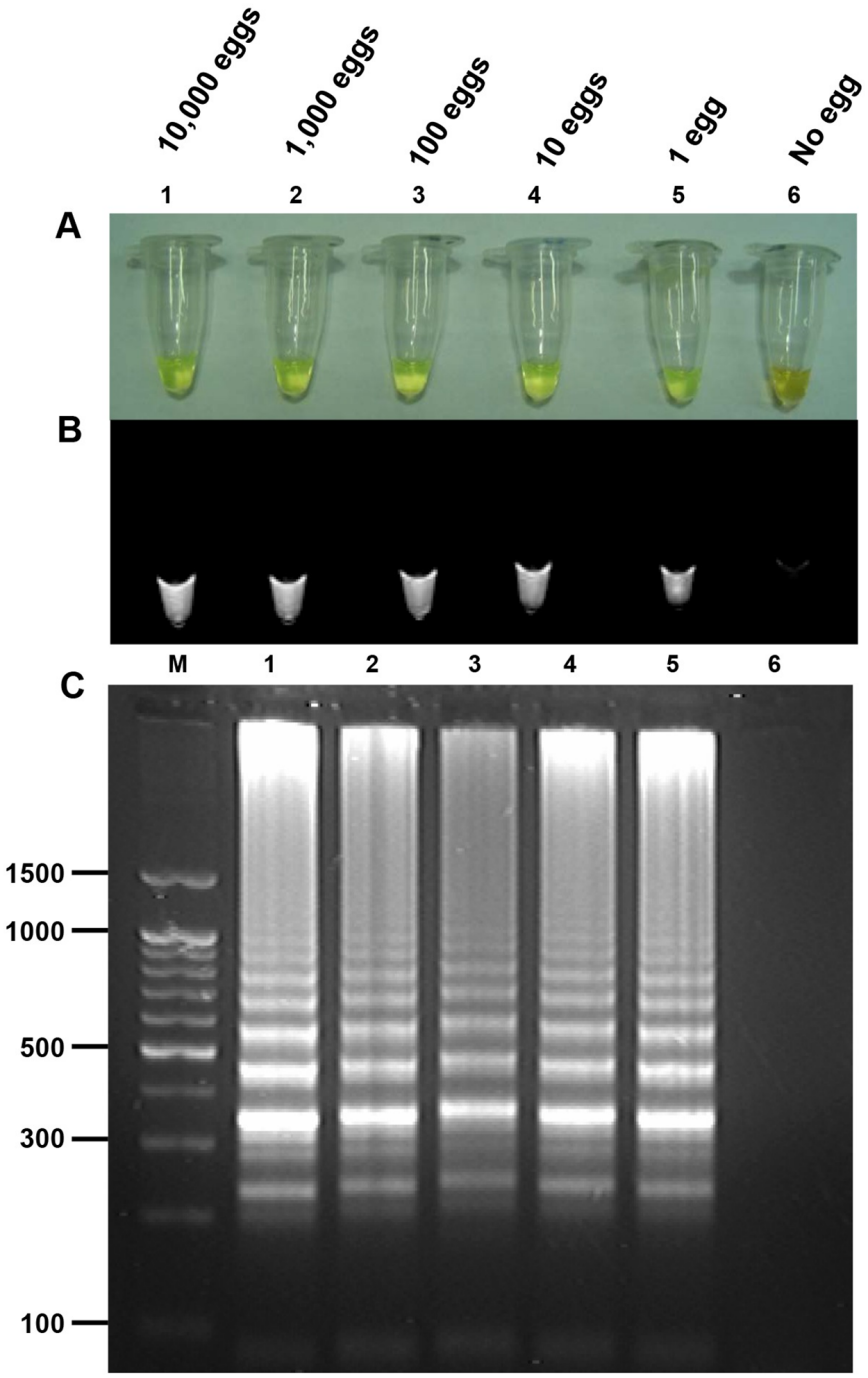
Sensitivity of the loop-mediated isothermal amplification (LAMP) assay for the detection of *Clonorchis sinensis* eggs in feces experimentally spiked with a known number of eggs in ten-fold serial dilutions from 10,000 eggs (lane 1) to 1 egg (lane 5). (A) Naked eye detection of LAMP products using SYBR Green I. A green color indicates a positive reaction, and an orange color indicates a negative reaction. (B) Fluorescence of LAMP products after using SYBR Green I followed by detection under UV light. (C) Agarose gel electrophoresis of LAMP products followed by ethidium bromide staining and detection under UV light. Values in the left are in base pairs. Lane 6, negative stool DNA; lane M, molecular marker.

**Fig 4:**
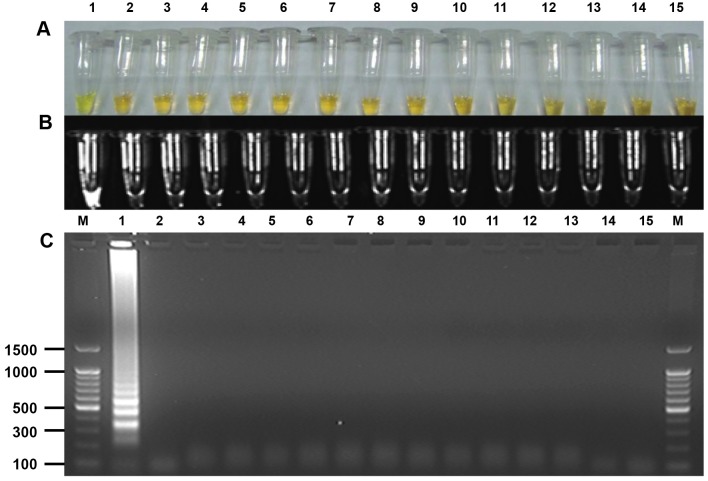
Specificity of the loop-mediated isothermal amplification (LAMP) assay for the detection of *Clonorchis sinensis* genomic DNA. (A) Naked eye detection of LAMP products using SYBR Green I. A green color indicates a positive reaction, and an orange color indicates a negative reaction. (B) Fluorescence of LAMP products after using SYBR Green I followed by detection under UV light. (C) Agarose gel electrophoresis of LAMP products followed by ethidium bromide staining and detection under UV light. Values in the left are in base pairs. Lane 1, *Clonorchis sinensis*; lane 2, *Metagonimus yokogawai*; lane 3, *Opisthorchis viverrini*; lane 4, *Fasciola gigantica*; lane 5, *Spirometra erinacei*; lane 6, *Diphyllobothrium latum*; lane 7, *Ascaris lumbricoides*; lane 8, *Ascaris suum*; lane 9, *Necator americanus*; lane 10, *Trichuris trichiura*; lane 11, *Cryptosporidium parvum*; lane 12, *Entamoeba histolytica*; lane 13, *Giardia lamblia*; lane 14, *Escherichia coli*; lane 15, non-template control; M, molecular marker.

To determine the detection limit of the LAMP assay, a 10-fold serial dilution of genomic DNA of the *C*. *sinensis* adult worm was amplified by LAMP. The assay detected as little as 100 fg *C*. *sinensis* genomic DNA (Fig 2A, 2B and 2C), whereas PCR amplified as little as 10 pg *C*. *sinensis* genomic DNA (Fig 2D). The assay also detected DNA from feces experimentally spiked with a series of known numbers of *C*. *sinensis* eggs; the minimum detectable number of eggs was one in 100 mg of feces (Fig 3). However, the LAMP assay did not amplify DNA from other helminths, protozoa or *E*. *coli* (Fig 4).

The diagnostic efficiency of the LAMP assay was evaluated using 120 stool samples, including 70 positive and 50 negative samples, confirmed by both KK and real-time PCR. The sensitivity and specificity of the LAMP assay relative to the combined result of KK and real-time PCR was 97.1% (68/70) and 100% (50/50), respectively (Table 1 and S1 File). When the LAMP results were compared with the KK results, the LAMP assay could detect *C*. *sinensis* DNA in 41 (100%) of 41 stool samples with EPGs more than 100. However, the LAMP assay detected *C*. *sinensis* DNA in 27 (93.1%) of 29 stool samples with EPGs less than 100 (Table 2). The lowest EPG detected by the LAMP assay was 24. The two samples that were positive using KK and real-time PCR, but negative by the LAMP assay, had an EPG of 12.

**Table 1:** Results of the loop-mediated isothermal amplification (LAMP) assay for the detection of *Clonorchis sinensis* DNA in 120 stool samples compared with the results diagnosed by Kato-Katz (KK) and real-time PCR.

	LAMP assay					
KK & real-time PCR	Positive	Negative	Sensitivity (95% CI)	Specificity (95% CI)	PPV, %	NPV, %
**Positive (n = 70)**	68	2	97.1 (90.1–99.2)	100 (92.9–100)	100	96.2
**Negative (n = 50)**	0	50				

PPV, positive predictive value; NPV, negative predictive value

**Table 2:** Amplification of *Clonorchis sinensis* DNA in 120 stool samples by loop-mediated isothermal amplification (LAMP) according to the infection intensity presented as eggs per gram of feces (EPG).

Intensity of infection (EPG^a^)	No.	LAMP positive No. (%)
**0**	50	0 (0)
**12–100**	29	27 (93.1)
**101–500**	22	22 (100)
**501–1000**	10	10 (100)
**>1000**	9	9 (100)
**Total**	120	68

^a^Determined by the Kato-Katz method.

## Discussion

Despite the development of various sensitive and specific molecular tools, clonorchiasis is still diagnosed by stool examinations [1]. However, the microscopic approach can miss lightly-infected cases, and differential diagnosis with other minute intestinal flukes is often difficult and requires expertise for accurate diagnosis [5]. Because extremely low burden cases are more prevalent in endemic areas in Korea, where extensive control measures are executed by health care workers, it is still a challenge to diagnose cases of clonorchiasis [1]. To advance more useful clonorchiasis diagnostic approaches, we have developed a LAMP assay and evaluated its diagnostic efficacy in human fecal samples. This is the first report on the detection of *C*. *sinensis* DNA in human fecal samples using the LAMP assay.

In this study, the LAMP assay was highly sensitive and detected as little as 100 fg of *C*. *sinensis* genomic DNA, which is 100 times more sensitive than the PCR performed using the external LAMP primers. The lower detection limit of LAMP assay than that of conventional PCR is consistent with other studies with *C*. *sinensis* [19, 27]. The high sensitivity comes from its ability to detect as few as six copies of DNA in the reaction mixture [17]. Another reason for this high sensitivity is that LAMP primers have been designed based on the mitochondrial gene (*cox1*) of *C*. *sinensis*, which is present in hundreds to thousands of copies per cell [31]. LAMP assay targeting the mitochondrial gene (NADH dehydrogenase subunit 1, *nad1*) of *O*. *viverrini* was also highly sensitive to detect as little as 100 fg of genomic DNA, which is the same as our developed assay for *C*. *sinensis* [21] but another study targeting ribosomal DNA (internal transcribed spacer, ITS1) of *O*. *viverrini* showed less sensitivity with a detection limit around 1 pg DNA/μl [22].

In spite of high sensitivity with genomic DNA, human stool samples are highly challenging for DNA amplification. Even after the DNA extraction, remaining contaminants and the presence of DNA polymerase inhibitors in fecal constituents can inhibit DNA polymerase [32]. The inhibition is considered to be associated with false-negative results of stool samples evaluated by PCR [15, 33]. Besides the inhibitors, stool samples contain egg stage only, thus successful disruption of the egg shell is required to give access to DNA [12]. Although LAMP assays to detect *C*. *sinensis* were already applied to various samples such as adult worms, metacercariae, infected fish muscle [19] and freshwater snails [27], it is noteworthy that we achieved successful application of LAMP assay for human stool samples in the present study.

Despite challenges mentioned above, our LAMP assay on stool samples spiked with known number of eggs revealed the minimum detection limit as low as one egg in 100 mg stool that corresponds to 10 EPGs, and this is the lowest detection limit that can be achieved in our experiment settings with 100 mg of feces. It is important to lower minimum detection limit down to 12 EPGs because the presence of one egg in 2 KK examinations will have a result of 12 EPGs. With low enough detection limit, our LAMP assay could achieve sensitivity as high as 97.1%, but not 100% due to the two false-negatives in the stool samples with an EPG of 12. The inability of the LAMP assay to detect these two stool samples might be due to the presence of low numbers of eggs. Eggs, if present in low number, may not be homogenously distributed in the stool and therefore, a sampling of the stool for DNA extraction might have missed the eggs leading to the false negative result. Less detection sensitivity in the stool samples with low infection intensity is in agreement with a previous study, in which real-time PCR for *C*. *sinensis* failed to detect 6 out of 70 samples with an EPG below 100 [14]. Due to limited amount of stools utilized for LAMP assay and the stochastic nature of egg distribution in a fecal sample, more experiments would be needed to determine the true detection limit of the LAMP assay in comparison with KK and real-time PCR.

Although there is no previous report on LAMP method for detection of *C*. *sinensis* from stool samples, there are few reports for detection of *O*. *viverrini*, and only one had evaluated its sensitivity compared with KK method [22]. The sensitivity of the LAMP assay was 100% for detection of *O*. *viverrini* DNA in egg positive stool samples. The amount of stool prepared for LAMP assay was twice as much as the amount we prepared for, and the less amount utilized in this study could led to less sensitivity in low infection intensity. The false negative result that LAMP showed in this study, is also expected for KK methods in such a low infection intensity where eggs of similar morphology from other intestinal flukes may exist. However, due to high specificity, the LAMP assay would be able to detect specifically *C*. *sinensis* from such samples. Further diagnostic performance of the test will be followed using unknown samples to optimize the assay for application in the real field condition.

The less sensitivity is tolerable because not all clonorchiasis cases especially the low worm burden cases would proceed to cholangiocarcinoma if untreated [34]. However, it is still important to identify and treat such low burden cases of clonorchiasis in those countries where the elimination program is in close proximity. Because patients with low EPG does not always represent the real situation of worm burden as they may harbor juvenile worms that might not have yet started laying eggs [35]. Application of this robust and highly sensitive LAMP assay for detection of such lightly infected cases in endemic countries would be really useful to make the controlling program success.

The sensitivity can be influenced by the volume of buffer used for final elution and the amount of template DNA added. Another study with stool samples utilized 50 μl of elution buffer on 200 mg of stool while this study used 100 μl of elution buffer on 100 mg of stool [22]. More densely eluted samples in the study can be one of the reasons for 100% sensitivity even in stool samples with less than 100 EPGs [22] in comparison with 93.1% sensitivity in the corresponding stool samples eluted by recommended methods in this study. Less elution buffer (50 μl) than the amount recommended by the manufacturer (200 μl) can increase the density of template DNA to help reaction [22], but, at the same time, can decrease the elution efficiency resulting in less sensitivity. Instead we increased the amount of template DNA to 4 μl to help the reaction. Prior to application to field samples, this kind of adjustment should be optimized using stool samples spiked by known number of eggs.

In addition, the choice of target genes can also influence sensitivity. As the amplification is sequence specific, the target genes should be conserved throughout the species. According to the investigation on DNA variations among three isolates of *C*. *sinensis* from Korea and China, few intraspecific nucleotide substitutions were found in 18S, ITS1, ITS2 and *cox1* sequences [36]. Among them, the nucleotide gap (insertion, deletion) differences were slightly larger for ITS1 [36], which showed less sensitivity than IST2 in PCR assays [15]. In this study, we designed the LAMP assay to target *cox1* gene, which shows very low level of intraspecific variation.

The specificity of LAMP is generally high because it uses four primers that recognize six locations on the target DNA [17]. In our study, in addition to four primers (two externals and two internals), two additional loop primers were included, resulting in recognition of a total of eight locations on the *cox1* gene of *C*. *sinensis*. Thus, the LAMP assay in the present study was highly specific and did not cross-react with the DNA of other helminths, protozoa or *E*. *coli* including the closely related liver fluke *O*. *viverrini*. Hence, the assay must be robust enough to specifically detect *C*. *sinensis* infection in areas where mixed parasite infections occur.

Despite inherent specificity of LAMP assay, the choice of target gene can influence the specificity as well. PCR assays targeting ITS regions of *C*. *sinensis* showed cross-reaction with the DNA of other liver flukes such as *O*. *viverrini* [12, 15] or *Opisthorchis felineus* [14]. PCR assays targeting other genes such as *cox1* and *nad1* have been developed for differential diagnosis of *C*. *sinensis* and *O*. *viverrini* [11, 13]. LAMP assays also encountered cross-reaction between *O*. *viverrini* and *O*. *felineus*, the researchers changed the target from ITS1 to microsatellites of *O*. *viverrini* and achieved the specificity [20]. Our target, *cox1* gene, did not cross-react with the DNA of other liver fluke *O*. *viverrini*.

The LAMP assay has several advantages in detecting *C*. *sinensis* DNA from stool samples compared to our previously developed real-time PCR assay [16] and commonly used conventional PCR. As the *Bst* DNA polymerase utilized for the LAMP assay is considered to be more resistant to the inhibitors [37], the LAMP assay have superiority for analyzing stool samples. In the aspect of efficiency, it can amplify large quantities of the target DNA under isothermal conditions with less time for DNA amplification [17]. Compared to the commonly used conventional PCR method, LAMP saves minimum 2 hours as it does not require thermocycler and gel electrophoresis. The cost of the LAMP assay is relatively less because it does not require sophisticated instruments like thermocycler for amplification of target gene, gel electrophoresis unit and gel documentation system for detection of amplified PCR products. A heat block or water bath is all that is required to perform the entire reaction successfully. Moreover, visual detection of the LAMP reaction is possible by simply adding fluorescent dyes, such as SYBR Green I, to the tube [18]. These features make the LAMP assay suitable for application to field laboratories in clonorchiasis endemic areas. Although the reaction of LAMP assay has been evaluated by naked eye with SYBR Green I, visualization under UV light, and agarose gel electrophoresis; in field condition visual judgment by naked eye with fluorescent dyes would be the appropriate approach of detection because it is achieved by simply adding dyes and does not require additional equipment or complicated procedures as we have demonstrated.

Longer time to read results and the requirement of equipment and materials for DNA extraction would prevent the use of LAMP assay at this level of development as a point-of-care diagnostic in low resource clinical settings. To enhance field applicability, more simple and user-friendly DNA preparations and a formulated ready-to-use reaction mixture should be available. Recently, a high throughput LAMP detection system has been developed for the diagnosis of malaria parasites which takes less than 2 hours from DNA extraction to reading results [38]. The system consists of a portable box which allows parallel processing of a large number of samples for DNA extraction and detection in such a way without the need of pipetting and centrifugation. In the future, a similar approach should be incorporated with our currently developed LAMP assay to promote the assay in poorly equipped laboratories.

In conclusion, we report a highly sensitive and specific LAMP assay for detection of *C*. *sinensis* DNA in human fecal samples. Due to the shorter reaction time and better visual judgment of positivity without requiring sophisticated instruments, the LAMP assay can be more easily applied in field laboratories than PCR as a powerful tool for more specific and reliable diagnosis of clonorchiasis, thereby improving both treatment and control programs.

## Supporting information

S1 File A flowchart showing experimental design and results. (PDF)

S2 File STARD checklist. (PDF)
