# Supplementary figures and images for: Application of a loop-mediated isothermal amplification (LAMP) assay targeting cox1 gene for the detection of Clonorchis sinensis in human fecal samples
Source: PLoS Negl Trop Dis. 2017 Oct 9;11(10):e0005995. doi: 10.1371/journal.pntd.0005995 (PMC5648269; doi:10.1371/journal.pntd.0005995)

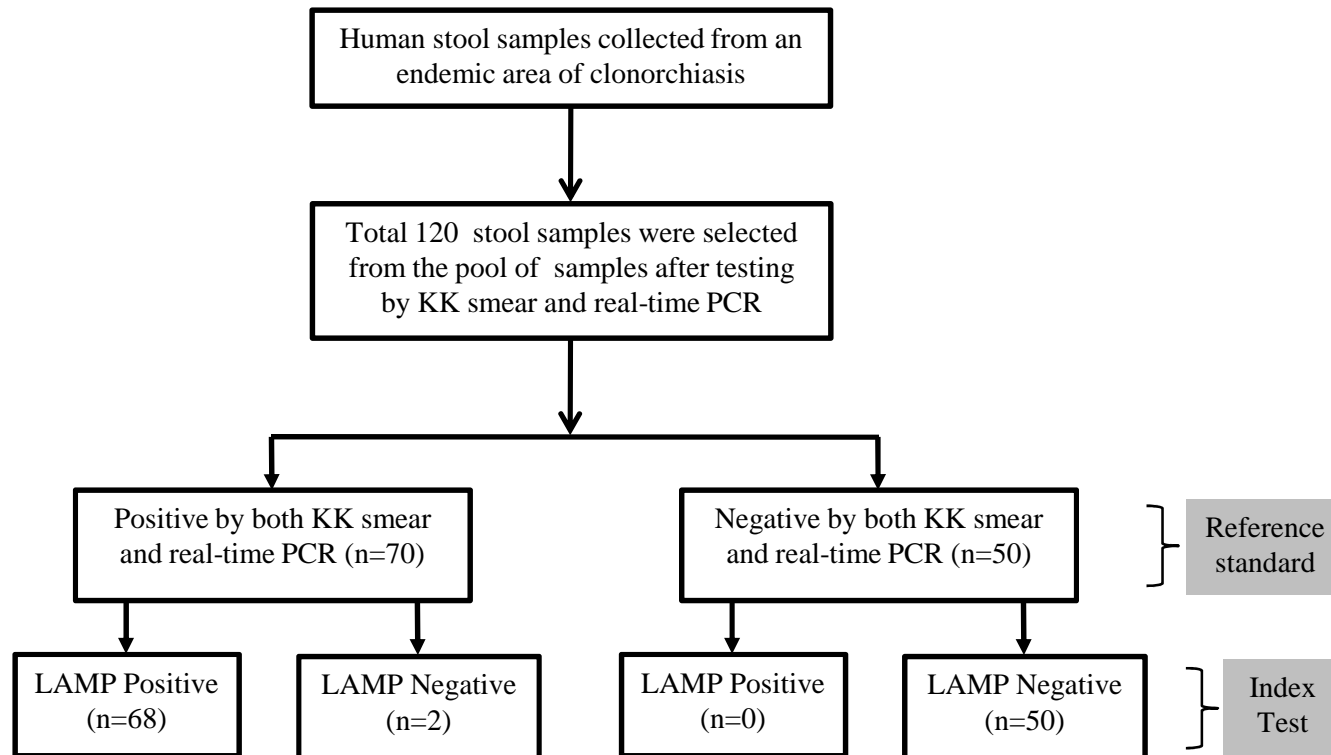

Supplement: S1 File — (PDF) [file pntd.0005995.s001.pdf]
